# A mass-market appraisal of the English housing rental market using a diverse range of modelling techniques

**DOI:** 10.1186/s40537-018-0154-3

**Published:** 2018-11-12

**Authors:** Stephen D. Clark, Nik Lomax

**Affiliations:** 1 Leeds Institute for Data Analytics, University of Leeds, Leeds, LS2 9JT UK; 2 School of Geography, University of Leeds, Leeds, LS2 9JT UK

**Keywords:** Housing, Rental, Regression, Machine learning, Big-data, Commercial

## Abstract

**Introduction:**

Mass appraisals in the rental housing market are far less common than those in the sales market. However, there is evidence for substantial growth in the rental market and this lack of insight hampers commercial organisations and local and national governments in understanding this market.

**Case description:**

This case study uses data that are supplied from a property listings web site and are unique in their scale, with over 1.2 million rental property listings available over a 2 year period. The data is analysed in a large data institute using generalised linear regression, machine learning and a pseudo practitioner based approach.

**Discussion and evaluation:**

The study should be seen as a practical guide for property professionals and academics wishing to undertake such appraisals and looking for guidance on the best methods to use. It also provides insight into the property characteristics which most influence rental listing price.

**Conclusions:**

From the regression analysis, attributes that increase the rental listing price are: the number of rooms in the property, proximity to central London and to railway stations, being located in more affluent neighbourhoods and being close to local amenities and better performing schools. Of the machine learning algorithms used, the two tree based approaches were seen to outperform the regression based approaches. In terms of a simple measure of the median appraisal error, a practitioner based approach is seen to outperform the modelling approaches. A practical finding is that the application of sophisticated machine learning algorithms to big data is still a challenge for modern desktop PCs.

## Introduction

This study is concerned with the operation of a mass market appraisal within the English housing private rental market [1] using a source of novel big data. Mass market appraisal is the ability to make an assessment of the potential rental value that a property can be listed at, using an automated approach with little or no intervention by rental professionals such as estate agents or letting agents [2]. The advantages of such approaches are that they are able to crunch through large volumes of information to provide these appraisals; they are based on an understanding of the current state of the market through the accumulation of information captured by novel data; and they can provide some insight into what is driving the market. Whilst the use of big data in the wider economy is advancing at a pace [3], application in real estate has been limited, with some notable exceptions [4, 5].

### Property market appraisals

Mass market sales appraisals are common and primarily needed for the levying of local property taxes [6–9]. These local taxes are usually used to fund local services as a supplement to either a local income tax or grants from regional and national governments. Since the market value of a property can only be truly determined when it is sold and then only for a period contemporary with this sale, external appraisals of house prices are periodically required. This ensures that such appraisals are consistent and fair in the locality and that each household makes the appropriate contribution, through the property tax base, to local services. The International Association of Assessing Officers [2] outline six broad approaches to making such appraisals: valuation models (usually built on a hedonic principle, [10]), cost models (based on the materials, design and labour used), use of comparable sales data (by matching the property with similar properties that have sold recently), income approaches (estimating the value of a property that the local labour market can sustain) or land value estimations (where land is a dominant cost associated with housing). For residential property sales they state that the comparable sales approach has been found to be efficient, supplemented by valuation models. The Zoopla property web site in the UK complements these approaches [11] with a method that takes the previous sales price of the property (where it exists) and applies a generic inflation/deflation figure to derive current valuations. Also, details of some recent methodological advances in automated appraisal methods can be found in d’Amato and Kauko [12].

### Rental market

Within the private rental sector there is less direct pressure for such mass market appraisals, although local property taxes are still usually levied on such properties so there is a need to ensure that such costs are covered through the rental charge. Instead there is the need to place a rental value on a property that reflects current market conditions. A rent too high and the property will remain on the market and not generate any income to the owner [13] and a too low rent will provide a deflated income to the owner [14]. Rental values are also useful in combination with sales values, where the rent–sales ratio provides an indication of the health, not only of the local housing market, but also the wider economy [15, 16]. Such appraisals need to take into account the structure and size of the property, the neighbourhood, the neighbourhood amenities and local environment [17].

In this study use is made of data obtained from an on-line database of rental listings supplemented by open data to provide 1-month ahead appraisals of the listed rental value of a diverse range of properties on offer in England. These appraisal are obtained using a sales comparison approach and their appraisal performance is compared with appraisals from a traditional hedonic model and an ensemble of machine learning approaches. The primary measures of goodness of fit will be the correlation between the asking rent and appraised rent, and the distributional nature of the errors in these appraisals, helping to ensure that the performance statistic is readily comprehensible to lay people and practitioners [18].

The intended audience for this study are researchers interested in the application of machine learning algorithms to big data and practitioners in the field of real estate. Smith [5] recognises that:

*Now, the rise of the “big data” concept may at last be setting the scene for a breakthrough for real estate… The analytical skills and experience to harness the data successfully are developing too, and market globalisation is serving to increase awareness of data best practice from different markets around the world.*

It is the spirit of encouraging this breakthrough that motivates this study.

In this study the reader is provided with a brief introduction to the literature in this field. This essential exercise enables the work to build on what is already known about this issue and provide an understanding of the range of approaches. It also identifies gaps that this study can help fill. The property and ancillary data used in the models is introduced. The models are then described and the indicative results discussed. The study finishes with a consideration of the findings of this study.

### Rental market valuations

In contrast to the sales market, the valuation of properties in the rental market has received little academic study [19, 20]. This has primarily been due to the lack of available data on such transactions, with sales price data having been the priority for data capture by Government and State authorities. However the increased use of web sites for listing rental properties [21] has made quality rental data available in sufficient volumes for analysis based on individual properties.

#### Traditional models

Most analysis of this market starts from, or provides a comparative base using, a hedonic relationship to explain rental value. This is basically a multiple linear regression (MLR) model relating the outcome of the rental value to attributes relevant to the property. These are commonly classified as structural (e.g. property type, number of bedrooms or bathrooms), locational (e.g. proximity to various services or travel times to destinations) or neighbourhood (e.g. local measures of affluence or crime). Some studies recognise that the rental value can be positively skewed and for modelling purposes apply a logarithmic or square root transformation prior to analysis [16, 22–26]. However, others prefer to retain the original scale of the rental price variable [27–30].

The source of data for such models is increasingly coming from commercial rather than government or administrative sources. Property rental listing websites have the capability of harvesting vast quantities of rental offerings [21] that can be coupled with potentially rich attribute and spatial information. This volume of data presents some challenges and opportunities. Recognising that such data are not always validated, prior to analysis, these data are sometime cleaned, for example by ignoring rental prices outside some range [27], trimming the top and bottom 1% of data [15] or removing statistical outliers [30].

The goodness of fit of such models is commonly assessed by a statistical measure, e.g. the R^2^ model diagnostic; a summary statistic of prediction errors, e.g. the root mean square error or the percentage error; a distributional summary, e.g. the proportion of properties whose percentage error of the appraisal is below some threshold; or property sector derived measures, e.g. the Coefficient of Dispersion (COD) or Price-Related Differential (PRD) [31]. If obtained from an administrative or commercial source, the volume of data also permits the estimation and prediction to be carried out on distinct sets of data [28, 30].

Beyond these standard statistical models, recent research has extended these analysis to incorporate recognition of the spatial structure of the data, citing Tobler’s First Law of Geography [32],

*Everything is related to everything else, but near things are more related than distant things,*

and also alternative modelling paradigms that allow for a flexible expression of the relationship between rental value and the attributes, such as machine learning.

In terms of the spatial aspects, the spatial error models (SEM) and spatial autoregressive models (SAR) are often used [33] and a third alternative spatial extension is Geographically Weighted Regression (GWR) [34]. There is considerable debate about which, if any, approach is best. Some argue that the inclusion of spatially varying attributes in the MLR can stand instead of an explicit spatial structure in the analysis (see Bourassa, Cantoni [35] in the context of house sales and the findings of McCord, Davis [30] in the Belfast rental market). In most cases however SEM and SAR models are only found to provide modest improvements in model diagnostics.

#### Machine learning

With the advent of increased computing power and data availability, artificial intelligence or machine learning algorithms (MLA) have begun to be used for understanding and predicting property prices. What such algorithms are able to do is to capture well any non-linearity in the relationship between rental prices and other information, without this being explicitly stated but learned by examination of the data. They also have the capability of dealing automatically with issues around multicolinearity, variable selection and identifying interactions [36].

Much of the work in this area is concerned with predicting the sales price of properties. Abidoye and Chan [37] provide a review of the applications of one form of MLAs, artificial neural networks, finding most studies using sales data with just two concerned with rental values. So, whilst Schernthanner, Asche [20] state that *“no study has been found estimating rental prices* via *machine learning methods*”, some studies do exist. Chen, Liu [19] use six MLAs to appraise rental values in a metropolitan area of China; Del Giudice, De Paola [38] use genetic algorithms to perform the same task in Naples, Italy; and Ng and Deisenroth [39] describe a mobile app that uses a MLA to guide prospective renters to areas of London that meet their rental requirements, particularly in regards to the cost of rents. A study of properties in Madrid, Spain, by del Cacho [40] used a data rich database of over 25k rentals from an on-line portal to examine the predictive performance of traditional hedonic models and a range of MLAs.

## Case description

The data for this study is collected by Zoopla [41], a large on-line property listing company and has been processed by data services company WhenFresh [42]. The data comes from the calendar years 2014 and 2015, with 652,454 listings in 2014 and 552,459 listings in 2015. Of these, some listings have no rental price or rental data information; some are from before 2014; some are duplicated; it includes listings from Wales; and this study is only concerned with properties with a weekly rental price of less than or equal to £10,000, leaving n = 1,063,419 (88%) usable listings. These are big data which are large in volume (a review by Abidoye and Chan [37] identified studies using orders of magnitude fewer data); it has velocity, in that the database underlying these data are augmented on a daily basis; the veracity is good, as commercial interests rely on its accuracy; and the variety is wide, capturing the salient features of rental property across the country (and these are additionally augmented in this study with locational characteristics).

### Property information

The property information retained is the listing rental price of the property, the property type [one of bungalow, detached, semi-detached, terraced, flat (apartment) or unknown] the number of bedrooms, bathrooms, reception rooms, the date the property was listed and was rented, how many page views the listing received and the postcode. Since the final agreed rental price is unknown, the listing price is used in this study. The listing date, the rental date and the number of page views were used to calculate a banded page visit intensity measure (views per day) to capture how popular each property was. The count variables (e.g. number of bedrooms) are expanded into a set of binary indicator variables with a limit of 6 or more bedrooms and 5 or more for bathrooms and reception rooms. Each variable also had an explicit binary variable to indicate if the information is unknown; so the number of bedrooms variable is coded into 7 variables (1, 2, 3, 4, 5, 6 or more and NA bedrooms). This approach allows for both a flexible non-linear relationship with rental price as the number of rooms increases and also the ability to make a prediction even when a piece of information is missing (NA).

### Neighbourhood information

Using the postcode information, additional information is attached to the property. Firstly a measure of the affluence of its neighbourhood using the ACORN classification [43] with the Group level being used here (the lower the category letter, the greater the affluence of the neighbourhood). This captures the attractiveness of an area through neighbourhood wealth. Secondly an indexed measure of the accessibility to assets that could be deemed to be healthy (e.g. hospitals, leisure services or green spaces) or hazardous (e.g. fast food and gambling establishments) [44, 45] is attached. This captures the attractiveness of a property through its environment. Next, a measure of access to services, namely education and transport is added. For education, the Office for Standards in Education (OFSTED) rating of the nearest primary (ages 3–11) and secondary schools (ages 12–16 plus) are used (the ratings are: outstanding, good, requires improvement or inadequate) [46]. OFSTED do not inspect schools in Wales and for this reason Welsh rental properties are removed. The distance to the nearest rail or underground station in kilometres is added from data provided by the Department for Transport [47]. The final piece of information is the distance of the local authority in which the property is located from the City of London (in kilometres), used to capture the large house price and rental gradient associated with London [1]. In practice, these two distance gradients were sharper the nearer to the station and the City of London, so a natural logarithm of these distances is used. These data are used by all three estimation methods.

### Estimation methods

This study uses a variety of methods to conduct the mass appraisal of the English rental market. One is a quassi Poisson generalised linear model (GLM) to account for the skewed distribution of the rental price and its possible over-dispersion. Then, a number of machine learning algorithms are used, primarily tree based (gradient boost (GB) [48], Cubist [49]) or specialist non-linear models (support vector machines (SVM) [50], multiple adaptive splines (MARS) [51]).

The procedure for fitting GB is described in this pseudo code [36] pp 203–208:
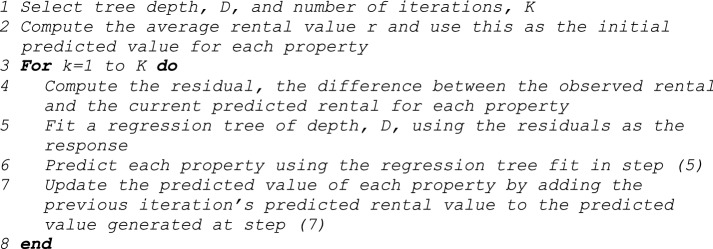


The procedure for Cubist involves the fitting of a regression tree and in addition the estimation of parameters for regression equations for those data that route through each note and leaf of the tree. Additional steps are taken to combine regression results from intermediate nodes; incentivise parsimonious regression equations; implement boosting to create ‘committees’ of predictions; and an optional phase to adjust using nearby observations [36] pp 184–190 and pp 208–212.

The procedure for SVM estimates the P parameters β that minimises:

$$cost \mathop \sum \limits_{i = 1}^{n} L_{\varepsilon } \left( {r_{i} - \hat{r}_{i} } \right) + \mathop \sum \limits_{j = 1}^{P} \beta_{j}^{2}$$

where cost is a scalar penalty set by the analyst; and L_ε_ is an e-sensitive function that sets values less than |1.0| to zero and values greater than |1.0| to their absolute value. This approach ensures that small residuals (< |1.0|) have no influence on the parameter; large residuals are not exaggerated; and large values for the parameters are penalised [36] p 151–159.

The final MLA are multiple adaptive splines which are estimated using a sequence of hinge functions:

$$h\left( x \right) = \left\{ {\begin{array}{*{20}c} {x \;\;\;x > 0} \\ {0 \;\;\;x \le 0} \\ \end{array} } \right.$$

in an equation of the form:

$$\hat{r}_{i} = \alpha + \beta_{1}^{A} h\left( {x_{1} - a} \right) + \beta_{2}^{A} h\left( {a - x_{1} } \right) + \beta_{1}^{B} h\left( {x_{2} - b} \right) + \beta_{2}^{B} h\left( {b - x_{2} } \right) + \cdots$$

See [36] pp 145–151.

All these methods are fitted within the machine learning paradigm of the caret package [52] in R [53].

The final method used is akin to a practitioner based approach (PBA), where the rental price is a summary (in this case an inverse distance weighted mean) of the rental price of recently rented similar properties in its neighbourhood, where here a practitioner would be an estate or letting agent [54]. For model training, the root mean square error (RMSE) calibration metric is used to select the optimal value for each model’s parameters.

### Experimental procedure

All these methods are applied in a consistent manner akin to a moving window. Prediction begins with the January 2015 listings. For this scenario, information from the previous 12 months, i.e. January 2014 to December 2014, is used to either appraise the relationship between rental price and various attributes or to form the database of similar properties. These relationships or database are then used to predict the out-of-sample January 2015 rental prices. Then prediction moves onto February 2015, using February 2014 to January 2015 data, and the final appraisals for December 2015 are based on December 2014 to November 2015 data. This approach allows for the adoption of the common within-sample training, out-of-sample testing paradigm used in machine learning and allows for models to be updated as new data becomes available, whilst less relevant historic data is removed.

#### Regression and machine learning

For the generalised linear models and machine learning algorithms a 1-times fivefold classification is used to establish the optimal parameters for the method. Whilst each 12 month training dataset will contain about 500k property listings, only a random subset of 5 times 50k are used for training and a separate 5 times 10k for testing (a total of 300k property listings). Once the optimal set of parameters have been established the final model will be trained on a large sample of 200k from the 500k. The prediction will be on the original scale of £, not the log transform scale (although this log transformation is implied for the GLM). From this exercise there are two parallel extensions. The first is to use the ensemble technique [55] to combine the predictions from these six MLAs using a linear model to attempt a more accurate prediction [19]. The second extension is to take the best performing MLA and re-run the training using all available historic data for final training, not just 200k; for each month this is around 500k data items. This will make this study one of the largest studies of a national, heterogeneous rental market ever undertaken.

#### Practitioner approach

For the PBA, to establish properties that are similar, they need to be: of the same property type, have the same number of bedrooms, bathrooms and reception rooms and be in the same ACORN Category. This later requirement is to ensure that the affluence of the neighbourhood is compatible amongst properties. Further options are available. Firstly there are thresholds to set for how far and how long ago rental listings should be used. Fuss and Koller [25] experiment with time windows of 400, 500 and 600 days and whilst there are differences in their model RMSEs, they are not great. Similar experiments with distance lags also suggest that their spatial models are largely invariant to these choices. Here time windows of 3, 6, 9 or 12 months (the scenario above is described using a 12 month time window) and distance lags of 15 km, 30 km, 45 km or 60 km are used. Secondly, given the filtering by these time and distance lags, the rental price is appraised using the inverse distance weight, with an index of 1, so that nearby properties “contribute more” to the appraisal than those further away. The final refinement is that when looking at similar properties, where there are more than 100 such properties any that are outliers are excluded from this calculation, much as a practitioner would discard untypical observations. The definition of an outlier here is an observation that is more than 1.5 times the inter-quartile range below the lower quartile or above the upper quartile (this is the standard definition of an outlier in a box-plot) thus ensuring that these limits are sensitive to the context of the data, e.g. higher limits for larger, more desirable, and expensive properties. Other studies arbitrarily trim the top and bottom 1% [15] or trim those that are more than 3 standard deviations beyond the mean [30].

There may be circumstances when there are no matching properties available within the time and distance thresholds set, in this case to guarantee an assessment a ‘national’ rental value table is used. This value is the median rental listing price in 2014 for similar properties irrespective of when they appeared on the market (i.e. the distance threshold is ignored and the time lag set to the whole of 2014 rather than the previous number of months). It is anticipated that these national assessments would be inferior, being less tailored to the more local conditions.

Algorithmically, this PBA is akin to a nearest neighbour approach, operating jointly in attribute space, geographic space and time. A general note here is that outliers are only removed in the ‘training’ data for the PBA, but when applying the PBA, estimates are made for all properties. Also for the other modelling approaches outliers are retained, since their training data would not routinely be subjected to any practitioner oversight—it is the algorithms task to deal with the presence of any outliers (see “Validation” section for the impact of this decision).

### Goodness of fit

In this study two measures of goodness of fit will be used, primarily driven by the need for such measures to be easily comprehensible and have been used in other studies to put these results in context. The first is simply the correlation squared between the observed rental price and the out of sample appraised price. This is described as the r^2^ statistic here. The second measure is the percentage error in the prediction, measured as the absolute difference between the listing price and the out-of-sample appraised price, divided by the listing price. This statistic is summarised as its median (the lower the median the better) and also as the proportion of these percentage errors below certain thresholds (the higher the proportion the better). These two measures are distinct from the RMSE calibration statistic and also readily comprehendible to the lay person.

## Results

To gain an understanding of the function of the English rental market a quasi-Poisson MLR models is run on the 2015 data. The results of the estimation are shown in Table 1 along with the sample size for each attribute. Measured against the 2011 Census table DC4407EW, these rental transactions during 2015 represent around 12% of the private rented stock in England. Only a proportion of this rental stock will be transacted in a given year and while there is no accessible administrative dataset recording the total number of transactions, the Valuation Office Agency [56] base their assessment of England’s private rental market on 439,599 entries in the lettings administrative information database for the 12 months to the end of March 2015. This analysis is therefore based on a similar sample size (n = 487,253) to that used by the VOA.

**Table 1:** GLM of 2015 rental market

Attribute	N/median	Estimate	Std error	t
Intercept	487,253	6.4510	0.0067	957.7***
Flat	212,275			
Bungalow	11,617	0.0073	0.0059	1.2
Detached	31,996	0.0192	0.0037	5.2***
Semi-detached	54,410	− 0.0463	0.0032	− 14.5***
Terraced	111,087	− 0.0185	0.0025	− 7.4***
Unknown	65,868	0.0169	0.0026	6.4***
1 bedroom	94,379			
2 bedrooms	192,236	0.2772	0.0024	116.8***
3 bedrooms	123,546	0.5157	0.0028	186.7***
4 bedrooms	41,505	0.7607	0.0033	228.6***
5 bedrooms	12,558	1.0080	0.0043	235.7***
6 and more bedrooms	7097	1.2650	0.0051	248.3***
Unknown bedrooms	15,932	− 0.0881	0.0050	− 17.7***
1 bathroom	194,157			
2 bathrooms	45,440	0.1314	0.0026	50.8***
3 bathrooms	6767	0.3343	0.0047	71.2***
4 bathrooms	1150	0.5347	0.0085	63.3***
5 and more bathrooms	622	0.6633	0.0107	62.0***
Unknown bathrooms	239,117	0.1169	0.0024	48.2***
1 reception room	159,999			
2 reception rooms	41,912	0.0020	0.0030	0.7
3 reception rooms	4921	0.0681	0.0060	11.4***
4 reception rooms	723	0.2235	0.0113	19.8***
5 and more reception rooms	191	0.3379	0.0189	17.9***
Unknown reception rooms	279,507	− 0.0333	0.0024	− 13.9***
January	50,988			
February	37,309	− 0.0220	0.0036	− 6.2***
March	39,601	− 0.0179	0.0035	− 5.1***
April	38,037	− 0.0098	0.0035	− 2.8**
May	40,414	0.0095	0.0034	2.8**
June	42,095	− 0.0090	0.0034	− 2.7**
July	44,808	− 0.0031	0.0033	− 0.9
August	39,791	0.0068	0.0035	2.0*
September	37,994	− 0.0041	0.0035	− 1.2
October	43,005	0.0086	0.0034	2.5*
November	42,037	0.0238	0.0034	7.0***
December	31,174	0.0042	0.0038	1.1
Up to 4 web site visits per day	24,094			
5–10 web site visits per day	14,610	0.0244	0.0055	4.4***
11–20 web site visits per day	23,114	− 0.0199	0.0050	− 3.9***
21–60 web site visits per day	39,969	− 0.0469	0.0046	− 10.3***
61 and more web site visits per day	29,423	− 0.0754	0.0050	− 15.2***
Unknown site visits	356,043	0.0230	0.0037	6.2***
Affluent achievers	60,017			
Rising prosperity	136,624	− 0.1961	0.0026	− 74.5***
Comfortable communities	98,779	− 0.2798	0.0028	− 99.7***
Financially stretched	92,146	− 0.3463	0.0031	− 112.9***
Urban adversity	96,472	− 0.4212	0.0031	− 134.3***
Not private households	3008	− 0.0994	0.0090	− 11.1***
ACORN not known	207	− 0.1028	0.0274	− 3.8***
Distance from the City of London (logged in model)	113.95 km	− 0.2862	0.00079	− 363.2***
Distance from railway station (logged in model)	1.11 km	− 0.0204	0.0010	− 20.0***
Outstanding primary school	91,869			
Good primary school	308,287	− 0.0487	0.0019	− 26.2***
Requires improvement primary school	79,841	− 0.0614	0.0026	− 24.0***
Inadequate primary school	7256	− 0.0972	0.0071	− 13.7***
Outstanding secondary school	119,014			
Good secondary school	245,070	− 0.0760	0.0018	− 43.2***
Requires improvement secondary school	96,715	− 0.1047	0.0024	− 44.6***
Inadequate secondary school	26,454	− 0.1269	0.0044	− 28.9***
Retail health	30.53	0.0025	0.00005	52.2***
Access health	7.21	− 0.0001	0.00008	− 1.9
Environment health	25.32	0.0004	0.00004	10.5***

*Note* Statistical significance: *** < 0.1%; ** < 1%; * < 5%; . < 10%

### Regression model

No r^2^ statistic is available for this model, but on the log scale the squared correlation between observed and in-sample predicted, r^2^, is 0.738, and on the original £ scale (using methods outlined by Duan [57] to transform back) r^2^ is 0.54. In terms of property type it appears that, all other things been equal, detached properties command a significant price premium in this market over other property types, with semi-detached the least desirable. The more bedrooms and bathrooms in a property, the higher the rental price. The increase for bedrooms is almost linear (+ 0.25 per bedroom). For reception rooms there is little price premium for 2 rooms over 1, but significant premiums for 3, 4 and 5 reception rooms. The pattern associated with the month of listing is less clear, May, August and the last 3 months of the year are those months where the listing price is significantly higher than in January, and otherwise the price is lower. The measure of intensity of web site visits shows a clear pattern for the properties with a high intensity of visit per day having a lower rental price. Living in neighbourhood where the residents are less affluent also lowers the rental price—the ACORN parameters follow the affluence gradient, more negative for less affluent areas. Being further from the City of London and further from a railway station lowers the rental price. As the quality of the local primary and secondary school diminishes, the rental price falls. Finally a neighbourhood with positive retail and environmental health increases the rental price, but access to actual health services is not significant. These outcomes all appear plausible, providing re-assurance that there is intelligence in these data, with the possible exception of property type.

### Machine learning and practitioner approach

Turning now to the alternative MLAs, the rolling window nature of the experimental procedure makes it is possible to examine appraisal performance month by month. For the machine learning algorithms the r^2^ performance during within sample training is shown in Table 2. The two tree-based methods, GB and Cubist, out-perform the regression based approaches of GLM, SVM and MARS. Full training sample Cubist (which is later seen to be the best MLA during testing) performs consistently with r^2^ between 0.63 and 0.67 and clearly performs best during training of all MLAs. There is no equivalent within sample training measure for the PBA, since all its predictions are out of sample. In terms of the computation for each month, SVM is by far the most time consuming, taking 1 h to estimate the optimum parameters using 50k of data during training and 5 h for the final fit using 200k of data. The other methods take between 1 and 1½ h per month, totalling 6 days of computational effort.

**Table 2:** Goodness of fit (r^2^) during training

Training	GLM	GB	SVM	Cubist	MARS	Best MLA
Jan	0.53	0.57	0.48	0.59	0.47	0.65
Feb	0.56	0.59	0.53	0.62	0.49	0.66
Mar	0.51	0.56	0.47	0.59	0.45	0.67
Apr	0.56	0.61	0.48	0.63	0.47	0.66
May	0.56	0.59	0.50	0.60	0.49	0.66
Jun	0.54	0.57	0.51	0.60	0.48	0.64
Jul	0.54	0.56	0.50	0.59	0.48	0.64
Aug	0.56	0.60	0.50	0.63	0.48	0.64
Sep	0.54	0.57	0.50	0.59	0.47	0.64
Oct	0.51	0.56	0.50	0.60	0.46	0.63
Nov	0.49	0.52	0.46	0.54	0.43	0.64
Dec	0.49	v54	0.47	0.57	0.43	0.63

**Fig. 1:**
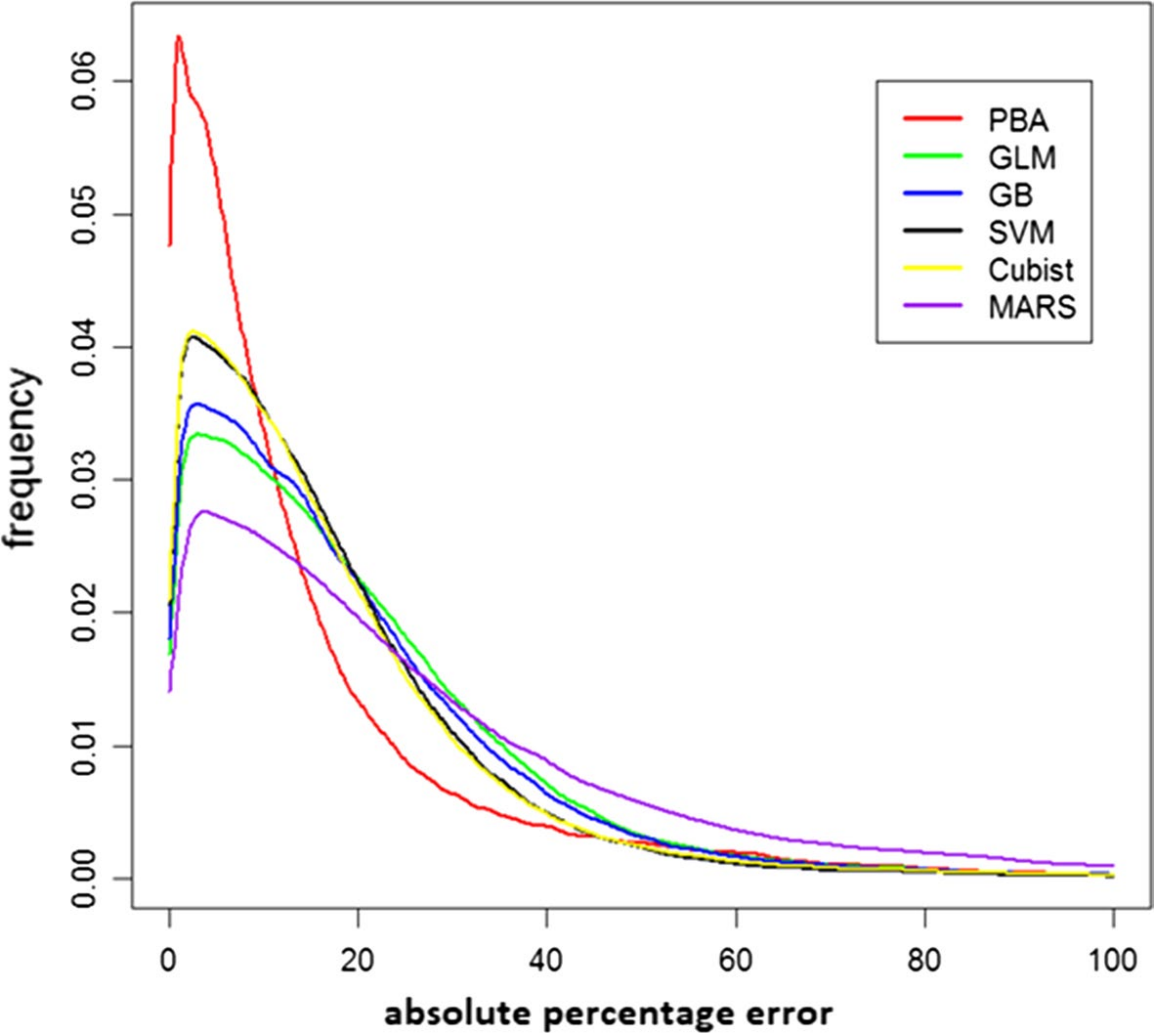
Distribution of absolute percentage errors

Turning now to the out of sample assessment performance, Tables 3 and 4 provide the goodness of fit measured as the r^2^ and the median percentage error. For the PBA model, a range of time horizons (3 months, 6 months, 9 months and 12 months) and distance thresholds (1 km, 2 km, 5 km, 10 km, 15 km, 30 km, 45 km and 60 km) are used and the combination that produces the lowest RMSE is 12 months with a 5 km distance threshold. With this combination, 85% of assessments were made with local data and the remaining 15% using the ‘national table’. By contrast, with a 12 months/60 km threshold, only 1% of assessments are based on the national table. There is a ‘reward’ associated with using fewer predictions from the national table—if all predictions were based on the national table, the median percentage appraisal error is nearly 25%. Notwithstanding this reliance on the national table, it is the 12 months/5 km is the version of the PBA that is used hereafter. For the r^2^ performance in Table 3, the best performing of the five machine learning algorithms is Cubist, followed by GB (and it is Cubist that is re-trained on the entire training data set—del Cacho [40] also found that the M5 algorithm, a close cousin of Cubist, performed well with their data). Ensembling the five MLA produced an improved goodness of fit marginally over Cubist, as does using all (approximately) 500k listings data during training for the moving window fits by Cubist, however the improvement does not match that seen during training in Table 2. The PBA performance is at best mediocre when compared to all the individual small training set MLAs and their ensemble and the best MLA trained on the full data set. All approaches predict poorly for the month of May. With the median percentage error in Table 4 the PBA out performs by some margin any of the MLA, with a lower median percentage error than the individual MLAs, their ensemble and the best MLA. To gain a further understanding of the distribution of these percentage errors, their distribution is plotted in Fig. 1. The reason why the PBA gives the lowest median percentage error in Table 4 is clear from this figure.

**Table 3:** Goodness of fit (r^2^) during testing/fitting

Testing	PBA	GLM	GB	SVM	Cubist	MARS	Ensemble	Best MLA
Jan	0.55	0.56	0.62	0.56	0.65	0.47	0.67	0.68
Feb	0.53	0.55	0.61	0.57	0.64	0.50	0.65	0.64
Mar	0.48	0.49	0.52	0.48	0.56	0.43	0.57	0.58
Apr	0.52	0.55	0.58	0.55	0.65	0.47	0.65	0.64
May	0.41	0.44	0.48	0.44	0.50	0.39	0.51	0.52
Jun	0.53	0.59	0.63	0.60	0.67	0.52	0.68	0.68
Jul	0.55	0.58	0.66	0.61	0.66	0.53	0.69	0.69
Aug	0.51	0.53	0.58	0.56	0.62	0.48	0.63	0.62
Sep	0.52	0.57	0.64	0.57	0.68	0.51	0.69	0.68
Oct	0.49	0.56	0.59	0.57	0.63	0.49	0.64	0.63
Nov	0.52	0.57	0.63	0.54	0.64	0.48	0.66	0.66
Dec	0.51	0.56	0.61	0.57	0.66	0.51	0.67	0.60
All	0.51	0.54	0.59	0.55	0.63	0.48	0.64	0.64

**Table 4:** Median percentage prediction error during testing/fitting (%)

Testing	PBA	GLM	GB	SVM	Cubist	MARS	Ensemble	Best MLA
Jan	7.95	16.62	16.07	13.80	13.59	20.73	13.44	13.28
Feb	8.17	16.55	15.22	13.30	13.46	20.66	13.04	13.02
Mar	8.35	16.28	15.24	13.32	13.22	20.66	13.14	12.89
Apr	8.47	15.83	15.00	13.13	13.31	20.49	12.95	13.05
May	8.62	15.94	14.85	12.99	13.04	20.01	13.32	12.98
Jun	8.82	16.02	15.07	13.39	13.36	19.83	13.04	13.13
Jul	9.23	15.68	14.82	12.97	12.91	19.69	12.87	12.57
Aug	9.26	15.70	14.74	13.02	12.90	19.92	12.91	12.74
Sep	9.26	15.12	14.40	12.55	12.38	19.25	12.40	12.31
Oct	9.80	16.14	15.17	13.40	13.39	19.67	13.39	13.10
Nov	9.95	16.70	15.76	13.83	13.89	19.64	14.46	13.36
Dec	9.73	15.77	14.76	13.20	12.35	19.36	13.00	13.03
All	9.07	16.04	15.11	13.25	13.18	20.01	13.06	12.95

**Fig. 2:**
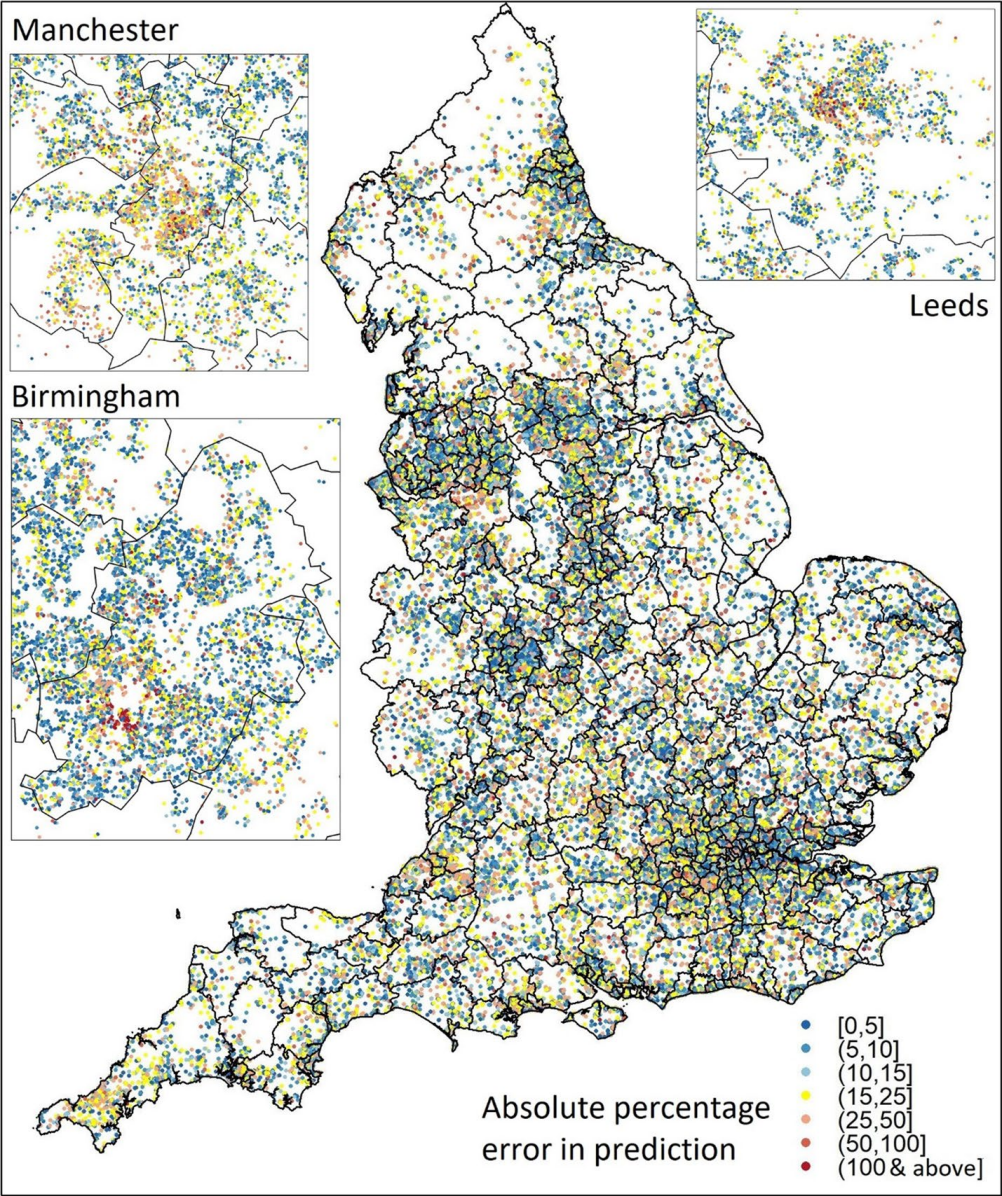
Absolute percentage prediction error from cubist model

To examine if there is any spatial structure to the absolute percentage errors, these are mapped by the properties location in Fig. 2. This map demonstrates that there is generally no sign of clustering (but see “Validation” section) and also the geographic scope and variety of the data used in this study.

## Discussion and evaluation

In this study a mass appraisal is performed for a significant proportion of England’s private rental market for 2015. A variety of approaches are attempted and in two of these approaches, a Cubist machine learning algorithm and the practitioner based approach, all the available, near 500k of data, is used for estimation. The appraisal performance of these models has been contrasting. The practitioner approach produced appraisals have much smaller percentage error whilst the other approaches have better r^2^. Clearly, which goodness of fit measure to take account of will depend on the circumstances, with a claim that a given proportion of properties with a predicted rental value within a percentage of the true value having some appeal [11].

### Comparative performance

Assessment of how the performance of these methods compares with those in similar studies of the rental market is difficult. Firstly there are few such comparative studies; secondly some researchers choose to model the log transform of the rental price (which would tend to produce better fits); thirdly there are a range of goodness of fit measures used; and finally sometimes predictions are made on the within data sample whilst in others the estimated model is used to predict for an out-of-sample dataset.

Taking r^2^ as a measure of goodness of fit, the highest r^2^ value found was that from Chung [58] who report an r^2^ of 0.98 (Table 3, Model 4) using log rental value as the dependant variable. For comparison, if the listing price and the out of sample predictions from the PBA model here are logged then the r^2^ increases from 0.50 to 0.76, still below Chung’s 0.98 value. Other reported r^2^ on the log scale are 0.329 and 0.315 in Appendix A of Banzhaf and Farooque [22]; 0.854 and 0.856 in Table 6 of Löchl [59]; Fuss and Koller [25] quote 0.883 (Table 3, STAR model), finally Baron and Kaplan [26] report 0.753 in Table 3. On the untransformed scale, Prunty [29] reports R^2^ of 0.19 for his California model and 0.13 for his New York model, Table 11; much higher R^2^ values of 0.607 and 0.622 are reported by McCord, Davis [30] in Table V. Clearly, even in this limited number of studies the range of R^2^ values is wide but these results sit comfortably within this range.

The comparison between the distributional aspects of the percent error in prediction are summarised in Table 5. Models on the log scale have better performance as do models evaluated on within-sample predictions. The log transformed practitioner model reported here is inferior to the SARerr model of Löchl [59] (although these are in sample) and superior to that of Fuss and Koller [25]. The original scale model is inferior to the results of McCord, Davis [30] (however these are again in-sample predictions).

**Table 5:** Comparison of distributional prediction performance

Scale	Log transformed			Original	
Source	PBA model	Löchl [59]	Fuss and Koller [25]	PBA Model	McCord, Davis [30]
Location	15 km and 12 months	Table 9, SARerr	Table 4/C, STAR	15 km and 12 months	
Testing data	1 month ahead	In sample	1 day ahead	1 month ahead	In sample
≤ 2%	54.69	72.65		15.1	13.3
≤ 5%	83.39	98.02	37.4	32.2	33.7
≤ 8%	91.85	99.93			
≤ 10%	94.42		64.8	53.3	60.9
≤ 15%	97.38		80.9	66.9	79.3
≤ 20%	98.66		89.3		

The performance of the PBA model is seen to be mid-range in comparison to the few other models in the literature. However, to properly gauge its performance the size of the task needs to be taken into account. Many of the better models achieve their good result by concentrating on just one city or locale, e.g. Zurich or Belfast, or one sub-sample of the local housing market, e.g. apartments. Here a model for the whole of the heterogeneous English rental market is formed. Also the size of the data sets used here is in contrast to these other studies, which use thousands of data items whilst this study used 10s and 100s of thousands. Perhaps the only study similar in scale to this is by Banzhaf and Farooque [22] who model nearly 250,000 properties in their log transformed hedonic model, and their R^2^ is much lower than those reported here. They also did not attempt the range of machine learning algorithm used in this study. Of the similar studies that do use machine learning, Chen, Liu [19] used nearly 330k rental records but these are aggregated to a geography of 3.5k residential quarters and Ng and Deisenroth [39] model house sales price using small geographic defined subsets (~ 2k) of the 2400k, information poor (only five attributes for each property), records available to them.

### Validation

**Fig. 3:**
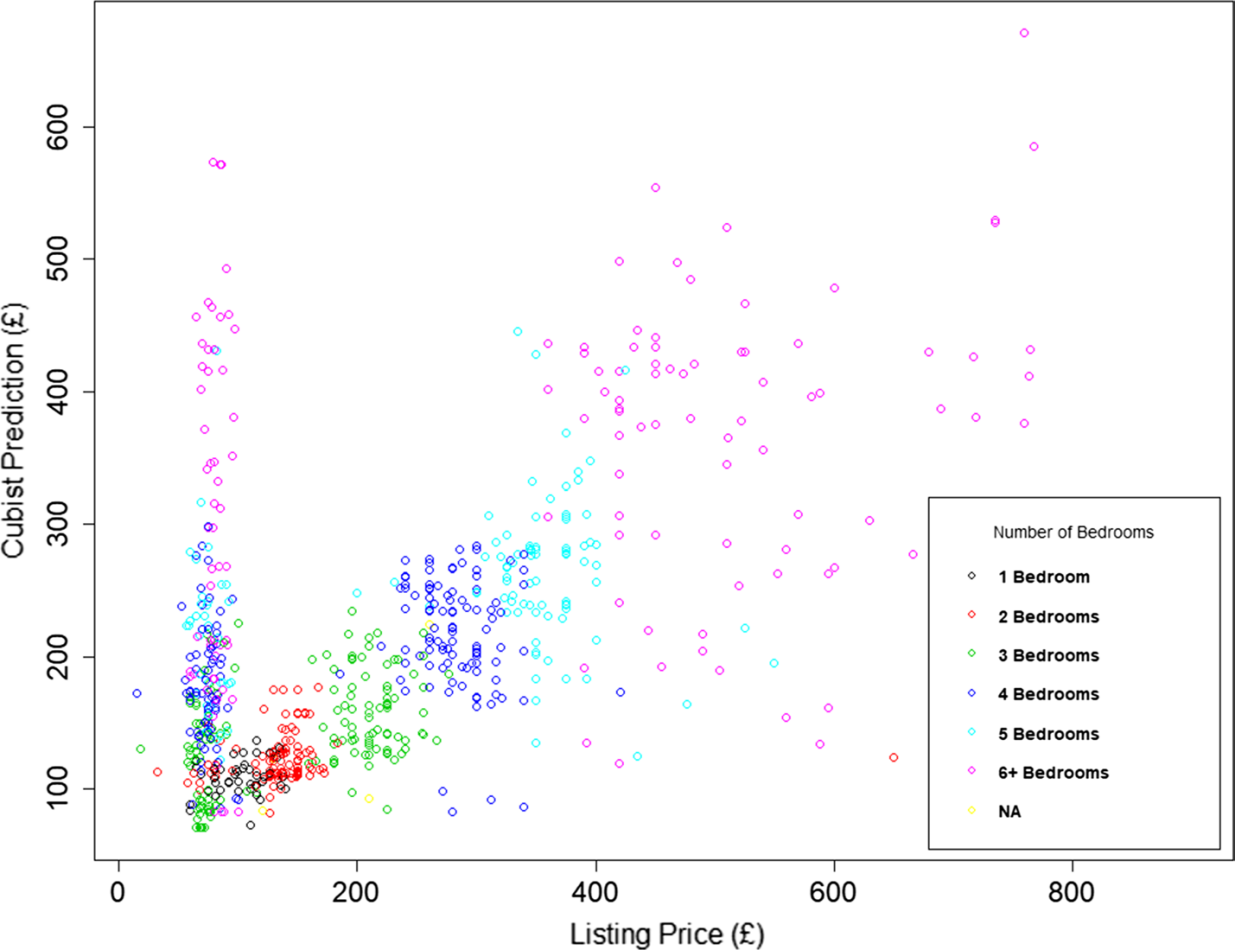
Listing and predicted list price for properties rented during 2015 in MSOA E02002383 in Hyde Park, Leeds

Ground truthing our results highlighted an issue which is apparent in some of the plots in Fig. 2. It is clear that there are clusters of properties where the prediction is poor, and local knowledge reveals many of these to be areas with a high proportion of student accommodation [60, 61]. By way of example, we look at an area called Hyde Park in the city of Leeds, (with a mid-2015 population of 8158) which is made up of densely packed terraced housing or low rise purpose built flats. During 2015, 792 properties were rented and the plot of the listing price and the prediction for these properties is shown in Fig. 3.

**Fig. 4:**
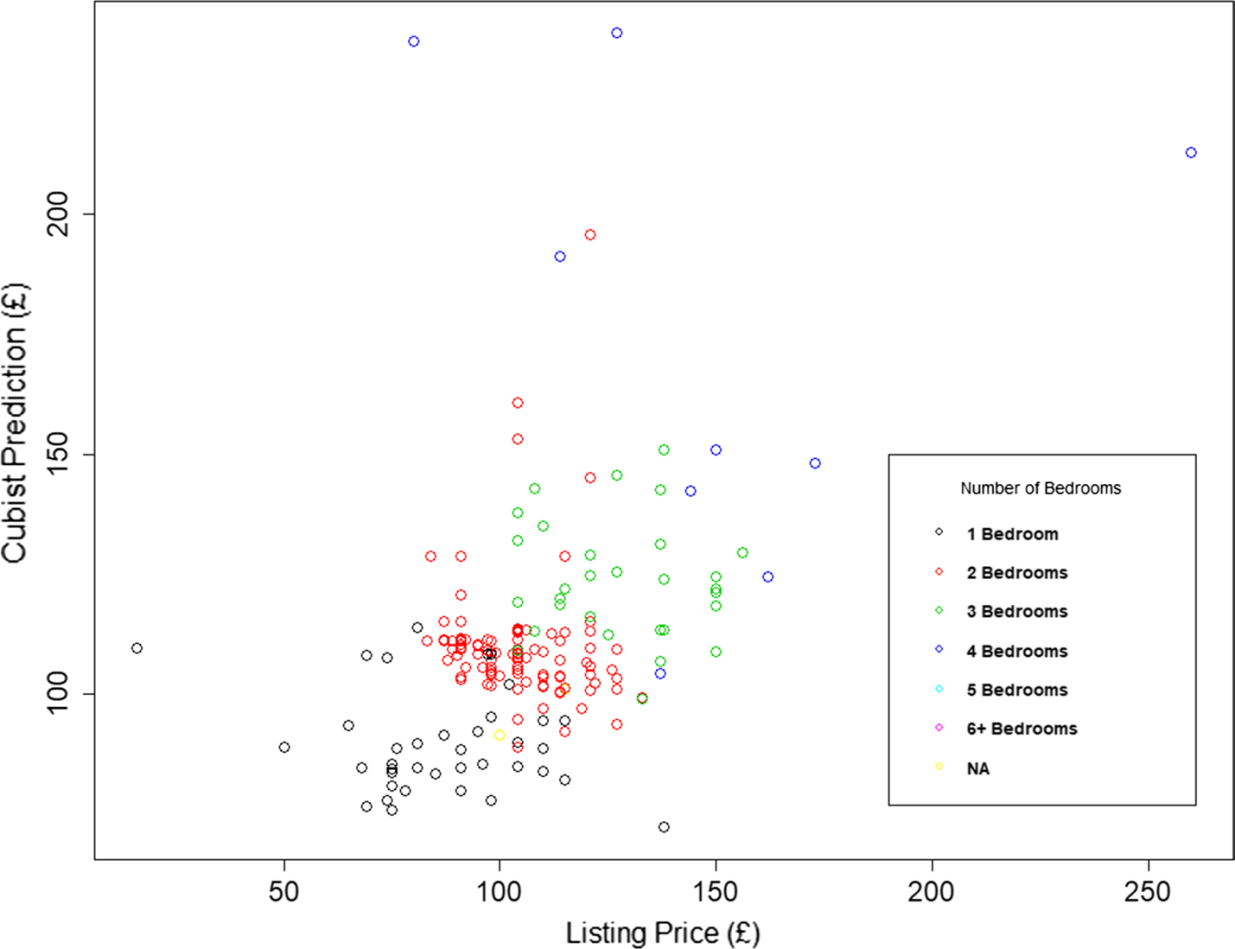
Listing and predicted list price for properties rented during 2015 in MSOA E02002400 in Armley, Leeds

This plot illustrates the issue that some properties have a low listing price but a high prediction. This is because, for a large number of student listings, the price quoted is for a room within a multi-room property, not for the entire property. In contrast, Fig. 4 shows the results for a nearby area of Leeds called Armley, which has similar housing stock to Hyde Park but a non-student resident population. Here there is no evidence of per room rentals. This example highlights that there is sometimes an inconsistency in what listing price represents. However, despite this anomaly, our models still perform well in predicting whole property values.

Early in this study a debate took place as to whether to ‘clean’ the data prior to its use in prediction. This was argued to be defensible in the case of the PBA, since this mimics human intervention which, when valuing a property, a listing that is clearly per room would be ignored or converted from a per room to a per property value by multiplying the listing price by the number of bedrooms. This is why outliers were removed in the PBA. However a more challenging approach was adopted with the MLAs, testing whether the intelligence in each algorithm would be able to cope with data that was either erroneous or incongruous—possibly contaminating the learning. This could be through an aspect of the data or the design of the algorithm. In the present context, the relevant information could be the ACORN classification (‘Student Life’ is a Group K in the ‘Financially Stretched Types’ Category number 4) or the particular seasonality when student properties tend to be let [62] (a third of the 792 properties are listed during November). From the results shown here in Fig. 3 it appears that Cubist has been robust to this inconsistency and able to estimate the listing price for these properties on a per-property bases—as required. It is then up to the users of these estimates to either list on this basis or the per-room basis.

### Limitations

Perhaps the most significant limitation to this study concerns the nature of the dependant variable, the property listing price. Ideally the actual price of the rental should be used, which would be more in line with market conditions. Unfortunately this actual price is not available from any source, as it is agreed between the landlord of the property and the eventual tenant but not routinely reported back to the property listing organisation. Even if this information was consistently available, commercial organisations may be reluctant to share it because it could undermine the desire to obtain the higher listing price for the organisation’s landlord client. Even so, knowing a competitive but realistic price to list the property at ensures that the property will be of interest to potential tenants and provides a reasonable starting point for any negotiations.

The next limitation is that for the range of five machine learning algorithms it has only been possible to use a sub-set of the available training data, constrained by the processing speed and memory capacity of even high-end desk top PCs. Other processing architectures will be needed to exploit even more of these data.

Finally there are some variables missing from these data which might improve performance. Two of these are the provision of parking spaces and whether the property is furnished. A property that has dedicated parking spaces and is furnished would likely command a higher listing rental price than would otherwise be the case. However in some contexts parking is not an issue, either being available for free on-street for houses, or generally not available, or available as a separate cost, particularly for city centre flats. In regards to furnishings, some studies have reported a significant negative effect on rental values when a property is partially or unfurnished, but McCord, Davis [30] in their study of the Belfast rental market find that there is no significant difference between furnished and unfurnished properties.

While there is a desire for more data to undertake analysis, nevertheless this study has utilised one of the largest property listing datasets, complete with property attributes, discussed in the literature. This has been combined with data on the local environment and level of amenity provision to create an attribute rich dataset with which has facilitated a market appraisal for transactions across the whole of England.

### Extensions

Both the MLR and MLA models allow predictions to be made on various scenarios. For example if a railway station was to open (or close) close to a property or the local primary school’s OFSTED rating should change, then both types of models are able to incorporate this change in circumstances and reflect this in the listing price. The ability of the PBA to reflect these changes is limited since its influential attributes are currently confined to the nature of the property and the affluence of the neighbourhood. However there is no reason why additional matching attributes cannot be incorporated into the PBA, but at the expense of producing potentially over specialised sub-markets.

If a comprehensive database of rental properties becomes available, say through a national census, that provides property attribute data comparable with these models, e.g. number of bedrooms, then such models can be used to gain a complete picture of the value of the English housing rental market. Another possible extension of this work is to apply methods outlined in this study to the house sales market—a companion data set on house sales from the same source as that used in this study is available and suitable for analysis.

As some of the references have shown, other countries are beginning to amass equivalent large databases of rental transactions (e.g. the USA [63], Australia [64] and the Netherlands [65]) meaning that the approaches to appraisal described here, particularly the novel practitioner based approach, are possible elsewhere—encouraging a drive to supplement traditional hedonic models.

## Conclusions

In this study a comprehensive mass appraisal of the English private rental market is reported. The study extends our understanding in a number of ways. Firstly it is an assessment for the transactions that occurred in the whole of the country of England, with many other previous studies being limited to geographic sub-markets such as cities or locals within cities, limiting the transferability of their findings. Secondly, it has recognised the heterogeneity of the English housing market, particularly in the sub-markets defined by the property type—again not being limited to just one sub-market, e.g. flats. It has also used contrasting approaches for the appraisal: a practitioner based approach, a hedonic model, and a range of machine learning algorithms. Often just the first two approaches are applied, although the collection of articles in d’Amato and Kauko [12] show that a variety of novel techniques are beginning to be considered, and if it is the predictive performance that is of most interest, machine leaning techniques need to be more fully considered by practitioners and academics. For some instances it has also highlighted the importance of sense checking the data prior to analysis so that the nature of the results can be understood. The final contribution comes from the large volume of data used, nearly 500k individual property transactions in combination with machine learning approaches. Commonly in other studies using these techniques, fewer data points than this are used or the data is aggregated to an administrative geography providing far fewer data points.

This study furthers the agenda of Smith [5] who argues that:

*Big data seek to combine processing power and specialist analytical skills to bring together huge, disparate and often incompatible data sets from different sources. If big data are to be “the next frontier for innovation, competition, and productivity” as the title of the McKinsey report* [66] *suggests, it would seem important for the real estate industry, and researchers in the sector, to identify areas where the value of harnessing big data outweighs the perceived advantages of keeping data private, and to start exploiting them.*

One challenge has been the limitations of modern high-end desktop PC hardware to apply some of the machine learning approaches in a reasonable amount of time. In future, the volume of novel data that becomes available can only increase in size and complexity, so it is important that algorithms and their implementations, particularly in regards to training, keep pace with this growth. However once trained or re-trained (the time consuming task) the application of the model in making predictions is quick (a matter of micro-seconds per property). This means that the outcome of interest, here the listing price, can be obtained almost instantaneously.

## Abbreviations

ACORN: an area classification system
BMA: Belfast metropolitan area
GB: gradient boost machine
GLM: general linear model
GWR: geographically weighted regression
MARS: multiplicative adaptive regression splines
MLA: machine learning algorithm
MLR: multiple linear regression
MSOA: middle layer super output area
NA: not available
OSTED: Office for Standards in Education
PBA: practitioner based approach
RMSE: root mean square error
SAR: spatial autoregressive model
SEM: spatial error model
SVM: support vector machines
